# Intravenous Administration of sRNA Nanoparticles for Treatment of Osteoporosis in Mice

**DOI:** 10.3390/pharmaceutics17060789

**Published:** 2025-06-17

**Authors:** Xuemeng Mu, Xinyi Du, Huitian Han, Fei Liu, Zhifa Zheng, Jing Hao, Lijin Liu, Su Liu, Ze Wei, Changfa Huang, Annan Liang, Wei Zou, Lina Zhao, Zhihong Wu, Jia Zhang

**Affiliations:** 1 Department of Orthopedic Surgery, Peking Union Medical College Hospital, Peking Union Medical College and Chinese Academy of Medical Sciences, Beijing 100730, China; 2 Department of Laboratory Medicine, Beijing Chaoyang Hospital, Capital Medical University, Beijing 100020, China; 3 State Key Laboratory of Complex Severe and Rare Diseases, Peking Union Medical College Hospital, Peking Union Medical College and Chinese Academy of Medical Sciences, Beijing 100730, China; 4 Stem Cell Facility, Institute of Clinical Medicine, Peking Union Medical College Hospital, Peking Union Medical College and Chinese Academy of Medical Sciences, Beijing 100730, China

**Keywords:** osteoporosis, sRNA delivery, lipid nanoparticles (LNPs), osteogenic differentiation

## Abstract

**Background**: With the intensification of population aging, osteoporosis has become one of the significant public health issues affecting human health. Currently available medications for treating osteoporosis are associated with various adverse effects and resistance issues. Oligonucleotide drugs show great potential. Effective delivery systems are essential to enhance the stability, bioavailability, and targeting of sRNA drugs. Lipid nanoparticles (LNPs) show promise as alternative osteoporosis therapeutics. This study explores the potential of LNPs as an effective delivery system to treat osteoporosis. **Methods**: LNPs were prepared using microfluidic techniques with varying lipid compositions, and characterized in terms of size, zeta potential, and entrapment efficiency (EE%). Dynamic light scattering (DLS) was employed to determine the size of the LNPs. The zeta potential was measured using electrophoretic light scattering. The pharmacodynamic effects and safety were then evaluated in a mouse model through intravenous administration. **Results**: Several lipid nanoparticle (LNP) formulations with different nitrogen/phosphorus ratios and different DMG-PEG2000 ratios were examined, and a lead candidate that supports delivery of sRNA in animal models of osteoporosis was identified. In OVX mice, LNP-sRNA significantly improved bone mineral density (BMD), trabecular microstructure, and biomechanical strength. Safety assessments revealed no systemic toxicity. It is shown that the optimized LNPs can serve as a promising delivery system to mediate sRNA delivery to bone tissue. **Conclusions**: After comparison of in vitro and in vivo properties, the optimized LNPs demonstrated good comprehensive performance as a delivery system for osteoporosis treatment. These results highlight the potential of the optimized LNPs as an ideal delivery system for osteoporosis, offering improved therapeutic efficacy and reduced systemic side effects.

## 1. Introduction

Osteoporosis is a systemic skeletal disorder characterized by reduced bone mass and microarchitectural deterioration, disrupting the balance between osteoblast-mediated bone formation and osteoclast-driven bone resorption, resulting in overactive osteoclasts [1,2]. With global population aging, osteoporosis represents a mounting public health concern [3].

Current osteoporosis treatments include anti-resorptive agents that inhibit osteoclastic activity and anabolic medications that stimulate osteoblastic bone formation [4]. Despite clinical efficacy, these treatments are limited by adverse effects, including gastrointestinal complications, osteonecrosis of the jaw, atypical femoral fractures, and cardiovascular risk with long-term use [5,6]. Anabolic agents such as parathyroid hormone derivatives face constraints due to short therapeutic windows and potential oncogenic risks [7]. These limitations underscore the need for novel therapeutic modalities [8].

Small nucleic acid drugs have become promising treatments due to their target specificity and ability to silence “undruggable” genes [9,10]. The clinical validation of sRNA therapeutics, exemplified by FDA-approved patisiran for hereditary transthyretin-mediated amyloidosis [11], has further accelerated interest in this approach for skeletal disorders. However, sRNA clinical translation for osteoporosis faces pharmacokinetic and biodistribution challenges, including rapid enzymatic degradation, inefficient cellular uptake, inadequate bone tissue specificity, and potential off-target effects [12,13]. The unique physiology of bone tissue—poorly vascularized and highly mineralized—further complicates targeted delivery [14,15].

Advances in RNA therapeutics have led to novel approaches for bone diseases. Jiang et al. pioneered identifying therapeutic RNAs from traditional medicines, introducing ‘Bencaosome’ as a platform integrating small RNAs derived from traditional Chinese herbal medicines with lipid-based delivery systems [16,17,18,19]. Building on this concept, we established a screening platform identifying bioactive small RNAs with therapeutic potential for bone diseases [20]. This study focuses on sRNA-OP-5, a 25-nucleotide single-stranded RNA that demonstrated osteogenic potential in preliminary evaluations. The study employs lipid nanoparticles (LNPs) optimized for sRNA delivery to bone tissue for osteoporosis treatment.

LNPs represent an established platform for nucleic acid delivery, as evidenced by their success in mRNA vaccines and FDA-approved sRNA therapeutics [21]. The core-shell structure of LNPs protects sRNA from nuclease degradation, prolongs circulation time, and facilitates endosomal escape following cellular internalization [22]. These versatile nanocarriers typically comprise four essential components: ionizable cationic lipids that complex with negatively charged nucleic acids, phospholipids that stabilize the lipid bilayer structure, cholesterol that enhances membrane rigidity, and polyethylene glycol (PEG)-lipids that provide steric stabilization and reduce opsonization [23]. Microfluidic manufacturing methods allow precise control over LNP size distribution, morphology, and internal structure [24,25].

While previous approaches for bone-targeted RNA delivery have primarily employed complex modifications such as bisphosphonate conjugation or hydroxyapatite-binding peptides [26,27], our study explores whether systematic optimization of conventional LNP parameters can achieve effective bone delivery without additional targeting moieties. This approach offers potential advantages in manufacturing simplicity, reduced immunogenicity, and enhanced clinical translatability. By carefully tuning the N/P ratio, PEG density, and microfluidic parameters, we aimed to develop an LNP formulation delivery to bone capable of simultaneously modulating both bone formation and resorption pathways.

Nucleic acid delivery for bone diseases faces challenges, particularly size-dependent extravasation through bone vasculature. Martin et al. [28] demonstrated that bone marrow vessels are hyperpermeable to nanomedicines in a size-dependent manner, with particles larger than ~30 nm facing extravasation barriers. Choe et al. [29] highlighted that even using targeted approaches with hydroxyapatite binding peptides, delivery efficiency to bone tissue remains a challenge. These studies underscore the importance of optimizing both size and surface properties of delivery systems for effective delivery to bone.

This study aims to develop and evaluate a LNP platform optimized through microfluidic techniques for intravenous delivery of therapeutic sRNAs targeting key regulators of skeletal homeostasis in osteoporosis. This study encompasses: (1) physicochemical characterization of LNP formulations; (2) assessment of systemic toxicity; and (3) evaluation of therapeutic efficacy in established osteoporosis models. This systematic approach addresses barriers in bone-specific sRNA delivery, establishing a foundation for next-generation anti-osteoporotic therapeutics.

## 2. Materials and Methods

### 2.1. Materials

DSPC (1,2-distearoyl-sn-glycero-3-phosphocholine), DOPE (1,2-dioleoyl-sn-glycero-3-phosphoethanolamine), cholesterol, DMG-PEG2000 (1,2-dimyristoyl-rac-glycero-3-methoxypolyethylene glycol-2000), and DLin-MC3-DMA (dilinoleylmethyl-4-dimethylaminobutyrate) were purchased from AVT Pharmaceutical Technology (Shanghai, China). Small RNA (sRNA) was custom synthesized by General Biology (Chuzhou, China). ALP quantitative kit, ALP staining, Oil Red O Staining Kit and CCK-8 assay were purchased from Beyotime (Shanghai, China).

### 2.2. LNP Formulation Details

#### 2.2.1. Lipid Nanoparticle Preparation

LNPs were synthesized via microfluidic mixing using different lipid molar ratios. Based on previous studies optimizing LNP delivery systems for short RNA molecules [30,31,32].

Three N/P ratios were evaluated (6:1, 8:1, and 10:1), as these ranges have shown effective complexation with small RNA molecules while maintaining appropriate particle stability [30,33]. For the lipid composition, we tested molar ratios of DLin-MC3-DMA:DSPC/DOPC:cholesterol:DMG-PEG2000 at 50:10:38.5:1.5 and 50:10:37.5:2.5, as these compositions have demonstrated effective endosomal escape and balanced circulation times in previous studies [31,34]. Microfluidic parameters were tested at flow rates of 8 mL/min and 12 mL/min based on established protocols for RNA encapsulation in LNPs [25,32].

The aqueous phase containing sRNA and lipid ethanol solution were mixed through the microfluidic mixer. Formulations were dialyzed against PBS (pH 7.4) for 24 h. The final LNP-sRNA formulation was filtered through a 0.22 μm sterile filter and stored at 4 °C until further use.

#### 2.2.2. Particle Size and Zeta Potential

Hydrodynamic diameter and polydispersity index (PDI) were measured using dynamic light scattering (DLS) and with Zetasizer Pro (Malvern Panalytical, Westborough, MA, USA). Zeta potential measurements were performed in 1 mM KCl using laser Doppler electrophoresis. Measurements were performed at 25 °C after appropriate dilution with ultrapure water. Each sample was measured in triplicate, and the results were expressed as mean ± standard deviation (SD).

#### 2.2.3. Entrapment Efficiency Percentage and Loading Efficiency

The entrapment efficiency (EE%) of sRNA in LNPs was determined using the Quant-iT RiboGreen RNA Assay Kit (Invitrogen, Carlsbad, CA, USA) according to the manufacturer’s protocol. LNPs were diluted 100-fold in TE buffer (10 mM Tris-HCl, 1 mM EDTA, pH 7.5) with or without 2% Triton X-100 to measure total and free sRNA, respectively. Fluorescence (Ex/Em: 480/520 nm) was quantified via a microplate reader, and EE% was derived by comparing values to a calibration curve of encapsulated sRNA standards. The efficiency was calculated as:

$$EE\%=\left(1-\frac{\left[Free\;siRNA\right]}{\left[Total\;siRNA\right]}\right)\times 100$$

### 2.3. In Vitro Evaluation

#### 2.3.1. Cytotoxicity Evaluation

Cells were purchased from the Peking Union Medical College Cell Culture Center. 293T cells were seeded in 96-well plates at a density of 1 × 10^4^ cells/well and cultured for 24 h. Cells were then treated with various concentrations of LNP-sRNA (0.0125~0.4 mg/mL) for 24 h. 10 μL of CCK-8 solution was added to each well and incubated for 2 h at 37 °C. The absorbance was measured at 450 nm using a microplate reader (BioTek Instruments, Winooski, VT, USA). Cell viability was calculated as a percentage relative to untreated control cells.

#### 2.3.2. RT-qPCR

Total RNA was extracted from cells or plasma using TRIzol reagent or TRIzol LS reagent according to the manufacturer’s instructions. RNA concentration and purity were determined using a NanoDrop 2000 spectrophotometer (Thermo Fisher Scientific, Waltham, MA, USA). First-strand cDNA was synthesized from 1 μg of total RNA using the PrimeScript RT Reagent Kit. RT-qPCR was performed using a LightCycler^®^ 480 device (Roche, Basel, Switzerland). The PCR conditions were as follows: initial denaturation at 95 °C for 5 min, followed by 40 cycles of denaturation at 95 °C for 10 s, annealing at 55 °C for 15 s, and extension at 72 °C for 20 s, then 72 °C for 10 min, eventually dropped to 40 °C. The primers used for qPCR were shown in Supplementary Table S1. Gene expression levels were normalized to Gapdh using the 2^(−ΔΔCt)^ method. All samples were analyzed in triplicate.

#### 2.3.3. ALP Activity Assay and ALP Staining

MC3T3-E1 cells were seeded in 12-well plates at a density of 5 × 10^4^ cells/well and cultured in osteogenic medium with various treatments for 3 days. For osteogenic differentiation, MC3T3-E1 cells were cultured in osteogenic medium containing α-MEM supplemented with 10% FBS, 10 mM β-glycerophosphate, 50 μg/mL ascorbic acid, and 10 nM dexamethasone. ALP activity assay kit and ALP staining kit were used to evaluate the content of ALP.

### 2.4. In Vivo Study

#### 2.4.1. Animals

6–8 weeks female C57BL/6J mice were purchased from the Vital River (Beijing, China). All mice were maintained under pathogen-free conditions under the rules of regulation. Animal care and surgical procedures were performed in accordance with the National Institute of Health’s Guide for the Care and Use of Laboratory Animals, with approval from the State Animal Welfare Committee (XHDW-2022-065).

#### 2.4.2. In Vivo Distribution of LNP-sRNA

Mice were administered LNP-sRNA via subcutaneous (SC) or IV injection using formulations with varying N/P ratios (6:1, 8:1, 10:1). Based on preliminary data, at 9 h post-injection, tissues (liver, bone, blood) were harvested, homogenized, and total RNA extracted. RT-qPCR (primers specific to sRNA sequence) was performed to quantify sRNA delivery efficiency (normalized to endogenous control genes). Optimal delivery route and N/P ratio were selected based on the results.

Using the optimal N/P ratio and administration method identified above, LNPs were prepared with different DMG-PEG2000 molar ratios (1.5%, 2.5%). Mice received IV injections (10 nmol sRNA), tissues were collected at 9 h, and sRNA levels analyzed by RT-qPCR.

LNPs with the optimal N/P ratio and DMG-PEG2000 ratio were synthesized at varying flow rates (8 mL/min vs. 12 mL/min). Delivery efficiency was assessed similarly via RT-qPCR to finalize the formulation.

‘Free uptake’ refers to administration of naked sRNA-OP-5 without LNP encapsulation, serving as a control to assess the delivery enhancement provided by LNP formulations. After testing different N/P ratios (6:1, 8:1, 10:1), flow rates (8 mL/min vs. 12 mL/min), and administration routes (SC vs. IV), we selected the N/P ratio of 8:1, flow rate of 8 mL/min, and IV administration for our optimized formulation based on the combined evaluation of physicochemical properties and tissue distribution results.

Cy7-labeled sRNA was encapsulated in LNP with the optimized formulation. 200 μL of Cy7-sRNA-LNPs (10 nmol sRNA) was administered to mice via IV injection. Ex vivo fluorescence imaging was performed on femurs at 9 h post-injection of Cy7-labeled LNP-sRNA or free Cy7-labeled sRNA.

#### 2.4.3. In Vivo Efficacy Studies—Osteoporosis Model

##### Model Development

The ovariectomy (OVX)-induced osteoporosis model was established in female C57BL/6J mice. Mice were randomly divided into four groups (*n* = 6): (1) Sham operation + PBS (Sham), (2) OVX + PBS (OVX), (3) OVX + LNP-sRNA (LNP-sRNA), and (4) OVX + Teripatatide (Teripatatide). Bilateral ovariectomy was performed under general anesthesia (intraperitoneal injection of 60 mg/kg pentobarbital sodium), while the sham operation involved exposure of the ovaries without removal. One week after surgery, mice received treatments for 5 weeks. OVX control groups did not receive injections. LNP-sRNA formulations (10 nmol sRNA) were administered via IV injection once a day, while teriparatide (100 μg/kg) was SC injected once a day. Body weight was monitored weekly throughout the experiment.

After 5 weeks of treatment, mice were euthanized, and the femurs were collected for micro-CT analysis, histological evaluation after decalcification, and biomechanical testing.

For the dose-dependent experiment: female C57BL/6J mice were randomly divided into six groups (*n* = 5): (1) Sham operation + PBS (Sham), (2) OVX + PBS (OVX), (3) OVX + LNP-sRNA (1 nmol), (4) OVX + LNP-sRNA (3 nmol), (5) OVX + LNP-sRNA (5 nmol), and (6) OVX + Teripatatide (Teripatatide). Other procedures were carried out as previously described.

##### Micro Computed Tomography (Micro-CT) Analysis

The femurs were collected, fixed in 4% paraformaldehyde for 24 h, and analyzed using a micro-CT system (μCT 100, SCANCO Medical AG, Zurich, Switzerland) with the following parameters: X-ray tube potential, 70 kV; intensity, 200 μA; integration time, 300 ms; resolution, 10 μm. The region of interest (ROI) was from the distal to the middle part of the femur. The following parameters were analyzed: bone mineral density (BMD), bone volume/tissue volume (BV/TV), trabecular thickness (Tb.Th), trabecular number (Tb.N), trabecular separation (Tb.Sp), and Ct. Th.

##### Three-Point Bending Test

The biomechanical properties of femurs were evaluated using a three-point bending test performed on a mechanical testing system (INSTRON E10000, Norwood, MA, USA). The bones were placed on two supports, and a load was applied to the midpoint at a rate of 3 mm/min until fracture. The maximum load (N) and max deflection (mm) were determined from the load–displacement curves.

##### Histology Analysis

The femurs were decalcified in 10% EDTA (pH 7.4) for 3 weeks, dehydrated through a graded ethanol series, and embedded in paraffin. Sections were cut and stained with hematoxylin and eosin (H&E) for histological analysis.

##### Analysis of Serum and Bone Tissues

The levels of cross-linked C-telopeptide of type I collagen (CTX-I) and osteocalcin (OCN) in the serum were determined using enzyme-linked immunosorbent assay (ELISA) kits (Cloud-clone, Wuhan, China) following the manufacturer’s instructions.

Total RNA was extracted from bone tissues, cDNA was synthesized and qRT-PCR was performed. Expression levels of osteoblast markers (*Alpl* and *Bglap*) were analyzed. β-Actin was used as an internal control. The relative gene expression was calculated using the 2^−ΔΔCT^ method. The primer sequences used for qRT-PCR are listed in Supplementary Table S1.

Calcium content in both serum and bone tissues was measured using Calcium Colorimetric Assay Kit (Beyotime, Shanghai, China) according to the manufacturer’s protocol. Results were expressed as mmol/L for serum and ug dry weight for bone tissue.

After euthanasia, femurs were carefully dissected from surrounding soft tissues. The length of each bone was measured using a digital caliper from the proximal to the distal end. Bone weight was determined using an analytical balance after the bones were cleaned and dried at room temperature for 24 h. Both bone length (mm) and weight (mg) were recorded for subsequent analysis.

#### 2.4.4. In Vivo Toxicity Evaluation—Acute Oral Toxicity Study

The acute oral toxicity of LNP-sRNA was assessed, dividing the mice into three groups (*n* = 3): (1) intrinsic drug-free LNP (Con), (2) LNP-NC-sRNA (NC-sRNA), and (3) LNP-OP-5-sRNA (10 nmol) (LNP-sRNA). The mice were monitored over 14 days for any toxic symptoms, changes in body weight, food consumption, behavioral alterations, adverse reactions, and mortality. After the 14 days observation period, the animals were sacrificed, and liver and kidneys were isolated for hematoxylin and eosin dyes to evaluate histological changes.

### 2.5. Statistical Analysis

All data were presented as mean ± standard error of the mean (SEM). Statistical analyses were performed using GraphPad Prism 8.0 software (GraphPad Software, San Diego, CA, USA). Comparisons between two groups were analyzed using Student’s *t*-test. Multiple group comparisons were conducted using one-way analysis of variance (ANOVA) followed by Tukey’s post hoc test. For in vivo studies with repeated measurements, two-way ANOVA with Bonferroni’s post hoc test was applied. Statistical significance was set at *p* < 0.05, and all experiments were performed at least in triplicate.

## 3. Results

### 3.1. sRNA-OP-5 Exhibits Anti-Osteoporotic Effects at the Cellular Level

**Figure 1:**
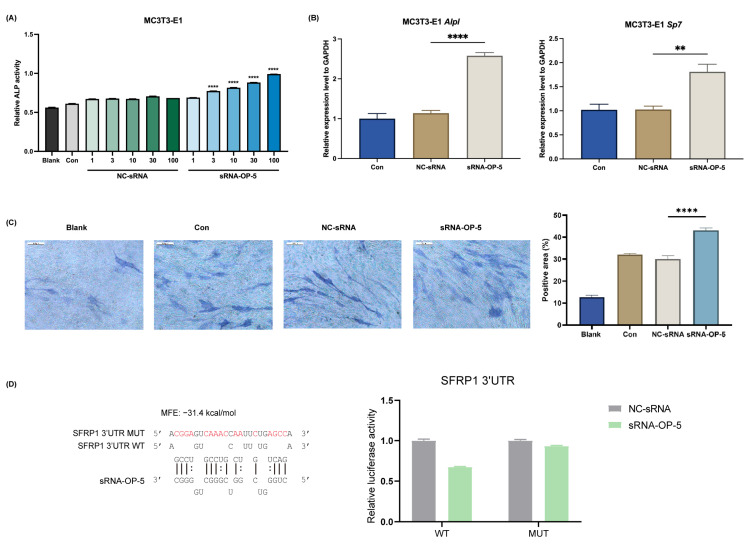
sRNA-OP-5 exhibits anti-osteoporotic effects at the cellular level. Cells were seeded in plates and allowed to attach overnight. The next day, when cell density reached 60–80%, sRNA was transfected at a final concentration of 50 nmol·L^−1^. (**A**) Dose-dependent effect of sRNA-OP-5 on relative alkaline phosphatase (ALP) activity in MC3T3-E1 cells compared to negative control sRNA (NC-sRNA) at concentrations ranging from 1–100 nM. sRNA-OP-5 treatment significantly increased ALP activity in a dose-dependent manner. (**B**) qRT-PCR analysis of osteogenic marker genes (*Alpl* and *Sp7*) expression in MC3T3-E1 cells after treatment with sRNA-OP-5 (100 nM) compared to control (Con) and negative control sRNA (NC-sRNA). Gene expression levels were normalized to GAPDH. sRNA-OP-5 treatment significantly upregulated the expression of osteogenic markers compared to control and NC-sRNA groups. (**C**) Representative ALP staining images (left) and quantification of positive staining area (right) in MC3T3-E1 cells treated with different conditions. sRNA-OP-5 treatment significantly enhanced ALP activity compared to all other groups. (**D**) Schematic illustration of the predicted binding site of sRNA-OP-5 to the WT and MUT SFRP1 mRNA. (**B**) Relative luciferase activity of the wild-type or mutant plasmids in HEK 293T cells. Luciferase activity was determined 48 h after transfection with 50 nM sRNA-OP-5 or NC-sRNA. Data are presented as mean ± SEM. ** *p* < 0.01, **** *p* < 0.0001.

As demonstrated in Figure 1A, sRNA-OP-5 treatment of MC3T3-E1 cells resulted in a dose-dependent increase in ALP activity, reaching approximately 200% of control levels at 100 nM concentration. The expression of osteogenesis-related genes was significantly upregulated. ALP staining provided further visual and quantitative evidence of enhanced bone formation potential (Figure 1C). sRNA-OP-5 simultaneously inhibited lipogenic metabolism (Figure S1). Reverse target prediction analysis of small RNAs indicated that sRNA-OP-5might target SFRP1 with an affinity of −31.4 kcal/mol. The binding between sRNA-OP-5 and sfrp1 was verified by the dual-luciferase reporter gene assay (Figure 1D).

### 3.2. Optimization of LNP Formulation Parameters

We adjusted the LNP formulation to enable more efficient delivery of sRNA-OP-5 to bone tissue.

**Figure 2:**
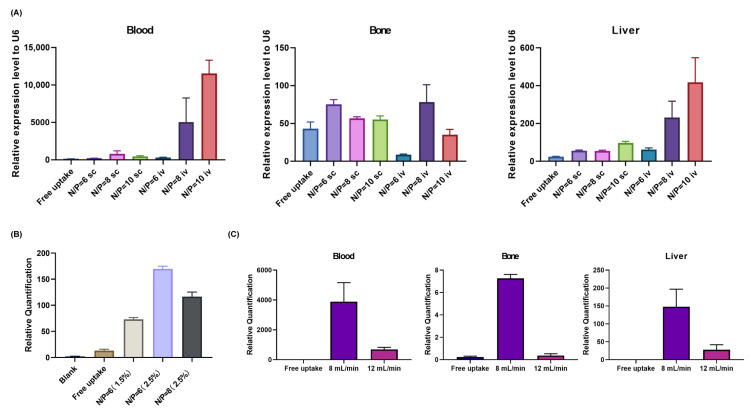
Optimization of LNP formulation parameters for effective sRNA delivery. (**A**). Effect of nitrogen/phosphorus (N/P) ratio on sRNA delivery efficiency to blood, bone, and liver tissues. The N/P ratio of 8:1 demonstrated superior delivery to bone tissue while maintaining balanced distribution in blood and liver compared to other ratios tested. (**B**). Influence of DMG-PEG2000 molar percentage on tissue distribution, showing enhanced bone delivery with 2.5% DMG-PEG2000 compared to lower concentrations, while maintaining appropriate blood circulation and liver distribution. (**C**). Impact of microfluidic flow rate ratio (FRR) on LNP distribution in blood, bone, and liver tissues. The flow rate of 8 mL/min yielded superior delivery efficiency compared to 12 mL/min, with appropriate distribution profiles in blood and liver. Free uptake controls at respective flow rates are shown for comparison. Data are presented as mean ± SEM, *n* = 3.

As shown in Figure 2 and Supplementary Tables S2–S4, we screened different nitrogen–phosphorus ratios, DMG-PEG2000 molar ratios, and microfluidic flow rates, and examined the particle size and encapsulation efficiency of LNP-sRNA-OP-5 (subsequently abbreviated as LNP-sRNA), as well as its tissue distribution after tail-vein injection into mice. The optimized LNP formulation consisted of DLin-MC3-DMA/DSPC/cholesterol/DMG-PEG2000 at a molar ratio of 50/35/10/2.5 mol%, prepared using a microfluidic flow rate of 8 mL/min and an N/P ratio of 8:1. This formulation demonstrated the best overall performance characteristics, including appropriate size (85.4 nm), low polydispersity index (0.15), high entrapment efficiency (94.4%), and a favorable tissue distribution profile with enhanced delivery to bone tissue. The optimized LNP formulation exhibited good stability. Storage at 4 °C for 7 days had almost no impact on the particle size and polydispersity index of LNP-sRNA (Supplementary Table S5).

### 3.3. Characterization and Biodistribution of Optimized LNP-sRNA Formulation

**Figure 3:**
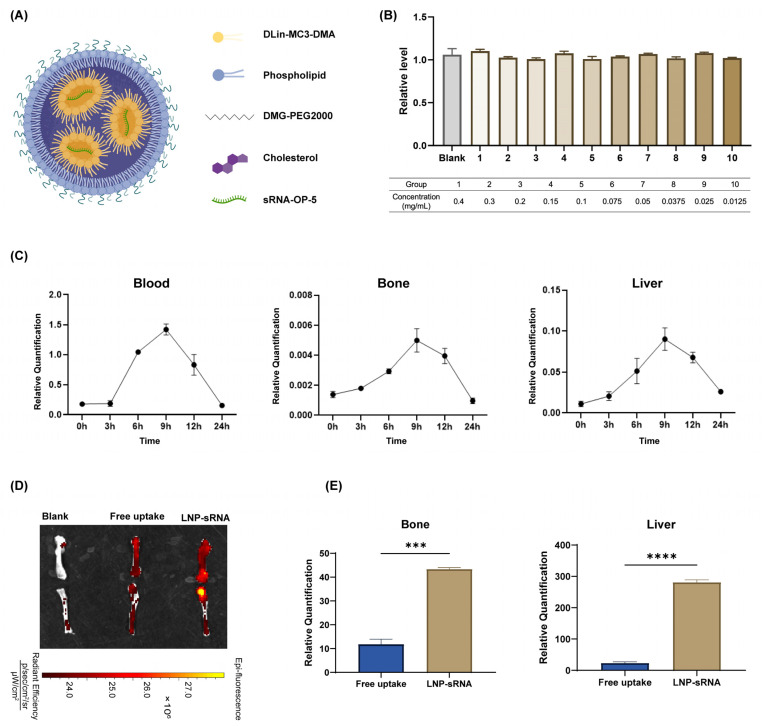
In vitro characterization and in vivo biodistribution of optimized LNP-sRNA formulation. (**A**). Schematic illustration of LNP-sRNA composition structure. (**B**). In vitro cytotoxicity evaluation of LNP-sRNA in 293T cells across various concentrations (0.0125–0.4 mg/mL), demonstrating high safety. (**C**). Time-dependent biodistribution analysis of optimized LNP-sRNA formulation. Relative sRNA expression levels in blood, bone, and liver tissues at different time points (0, 3, 6, 9, 12, and 24 h) following IV administration, quantified by RT-qPCR. Peak accumulation was observed at 9 h in all tissues. (**D**). Ex vivo fluorescence imaging of femurs 9 h after IV administration of free Cy7-labeled sRNA or Cy7-labeled sRNA encapsulated in optimized LNP formulation. The images demonstrate enhanced accumulation of LNP-sRNA in bone tissue compared to free sRNA. (**E**). Quantitative analysis of relative sRNA expression levels in bone and liver tissues at 9 h post-administration, indicating appreciable bone tissue penetration of the optimized LNP-sRNA formulation compared to free sRNA. Data are presented as mean ± SEM, *n* = 3. *** *p* < 0.001, **** *p* < 0.0001.

Figure 3A shows the composition of LNP components. We further examined the toxicity of LNP-sRNA. LNP-sRNA did not exhibit cytotoxicity in the 293T cell line.

In vivo biodistribution studies indicated that sRNA levels peaked at 9 h post-administration in blood, bone, and liver. The optimized LNP was capable of delivering sRNA to bone tissue. This finding was further verified by ex vivo fluorescence imaging of mice and RT-qPCR experiments. Compared with free uptake (naked RNA without any delivery vector), LNP-sRNA could deliver more RNA to bone tissue (Figure 3). LNP-sRNA could be applied to validate the subsequent mouse model of osteoporosis.

### 3.4. Therapeutic Efficacy in OVX-Induced Osteoporosis Model

The therapeutic potential of LNP-sRNA (10 nmol) was evaluated in an ovariectomy (OVX)-induced osteoporosis mouse model.

**Figure 4:**
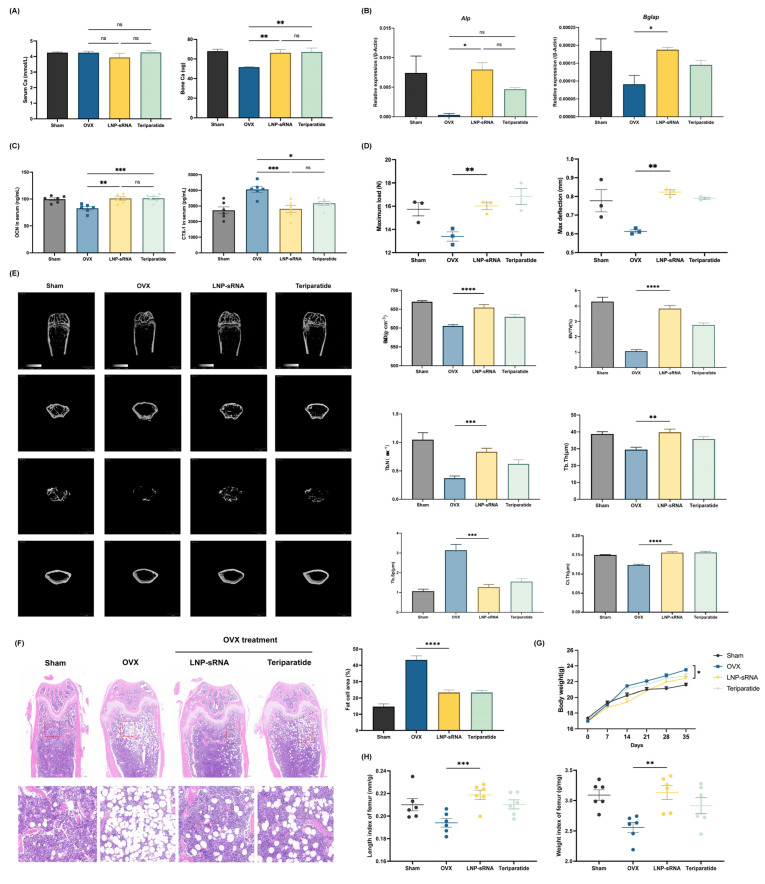
Therapeutic efficacy of LNP-sRNA in ovariectomy (OVX)-induced osteoporosis mouse model. (**A**). Bone and serum calcium levels across treatment groups. LNP-sRNA treatment significantly increased bone calcium content without affecting serum calcium levels. (**B**). RT-qPCR analysis of osteoblast marker genes (*Alpl* and *Bglap*) expression in bone tissue, showing enhanced osteogenic activity with LNP-sRNA treatment. (**C**). Serum bone turnover biomarkers measured by ELISA. LNP-sRNA treatment significantly reduced CTX-I (bone resorption marker) compared to the OVX group, and enhanced osteocalcin (bone formation marker) compared to the OVX group, with efficacy comparable to the positive control teriparatide treatment. (**D**). Biomechanical properties assessed by three-point bending test. LNP-sRNA treatment significantly improved maximum load and max deflection compared to OVX. (**E**). Representative micro-CT images (left) and quantitative analysis (right) of femur trabecular and cortical bone including BMD, BV/TV, Tb.Th, Tb.N, Tb.Sp, Ct.Th. LNP-sRNA treatment significantly improved both trabecular and cortical bone parameters compared to the OVX group. (**F**). Representative H&E staining of femur sections (left) and quantification of fat cell area percentage (right). The OVX group exhibits increased marrow adiposity, while LNP-sRNA treatment effectively inhibited adipogenic differentiation and promoted osteogenic differentiation, resulting in significantly improved bone microstructure compared to the OVX group. (**G**). Body weight changes monitored over the 35-day treatment period. LNP-sRNA treatment moderated OVX-induced weight gain. (**H**). Femur morphometric measurements including length and weight of femurs. LNP-sRNA treatment restored bone length and weight to near-sham levels. Data are presented as mean ± SEM. * *p* < 0.05, ** *p* < 0.01, *** *p* < 0.001, **** *p* < 0.0001; ns, not significant.

Bone calcium content was significantly increased in the LNP-sRNA treatment group, while serum calcium level was not significantly affected (Figure 4A). LNP-sRNA-treated mice showed increased mRNA levels of *Alpl* and *Bglap* in bone tissue, related to the osteogenic process (Figure 4B). Analysis of serum bone metabolism biomarkers using ELISA revealed that LNP-sRNA treatment significantly reduced bone resorption (decreased CTX-I levels) while concurrently enhancing bone formation (increased osteocalcin levels) (Figure 4C). This dual-action mechanism represents a significant advantage over conventional anti-resorptive therapies that primarily inhibit bone resorption without stimulating new bone formation [35].

Biomechanical testing (Figure 4D) demonstrated that LNP-sRNA treatment significantly enhance both maximum load and max deflection of femurs compared to the OVX group.

Micro-CT analysis (Figure 4E) demonstrated that LNP-sRNA treatment effectively preserved bone microarchitecture, significantly improving key parameters including BV/TV, Tb.Th, Tb.N, and BMD while reducing Tb.Sp compared to the OVX group. In addition to the trabecular bone improvements, micro-CT analysis revealed significant enhancement of cortical bone parameters in the LNP-sRNA treatment group. Cortical thickness (Ct.Th) was increased in LNP-sRNA-treated mice compared to the OVX group (*p* < 0.01). The cortical bone improvement aligns with the enhanced biomechanical properties observed in the three-point bending test, as cortical bone is the primary determinant of whole-bone mechanical strength.

Histological evaluation (Figure 4F) further confirmed these findings, with LNP-sRNA treatment effectively increasing the trabecular bone structure and decreased the proportion of adipocytes.

Body weight monitoring (Figure 4G) showed that OVX mice exhibited significant weight gain compared to the sham group throughout the treatment period, consistent with the known effects of estrogen deficiency on body composition. Treatment with LNP-sRNA attenuated this weight gain. Teriparatide treatment showed a similar trend to LNP-sRNA in moderating the OVX-induced weight gain. In addition, compared with the sham-operated mice, the femur length and weight of OVX mice decreased, and this downward trend was improved in the LNP-sRNA treatment group (Figure 4H).

**Figure 5:**
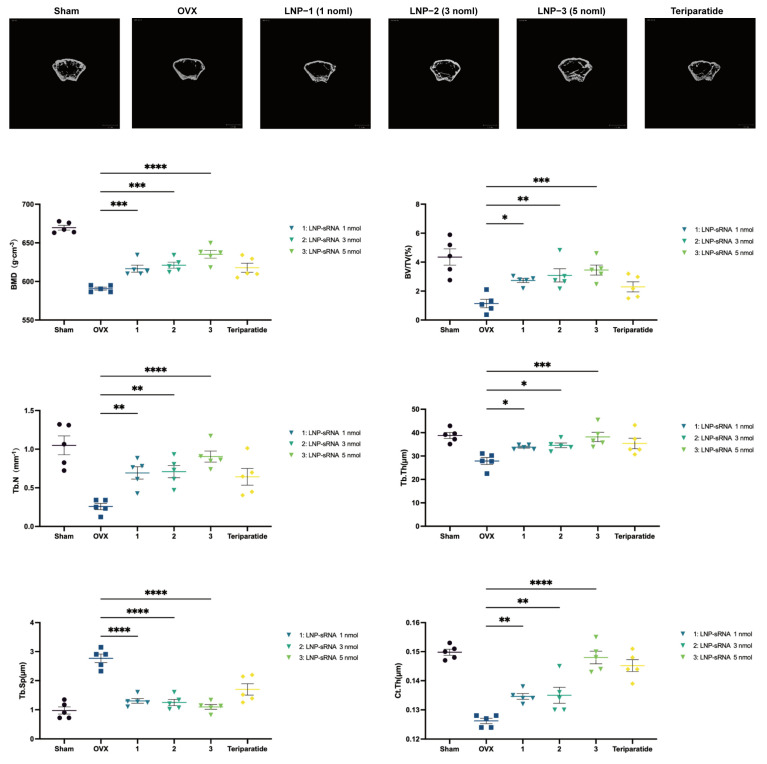
Dose-dependent effects of LNP-sRNA on bone microarchitecture in OVX-induced osteoporosis model. Upper panels: Representative micro-CT 3D reconstructed images of femurs from different treatment groups: Sham, OVX, LNP-1 (1 nmol), LNP-2 (3 nmol), LNP-3 (5 nmol), and Teriparatide. Images show cross-sections at different regions of the femur, demonstrating the progressive improvement in bone microstructure with increasing LNP-sRNA doses. Lower panels: Quantitative analysis of bone microarchitecture parameters including BMD, BV/TV, Tb.N, Tb.Th, Tb.Sp, and Ct.Th: All three doses of LNP-sRNA significantly improved bone microstructural parameters compared to the OVX group in a dose-dependent manner. Data are presented as mean ± SEM, *n* = 5. * *p* < 0.05, ** *p* < 0.01, *** *p* < 0.001, **** *p* < 0.0001.

The anti-osteoporotic effect of LNP-sRNA is dose-dependent; 1 nmol of LNP-sRNA can enhance bone mineral density and improve bone microstructure in OVX mice, indicating that LNP-sRNA has promising therapeutic potential (Figure 5).

### 3.5. Safety Profile of LNP-sRNA

**Figure 6:**
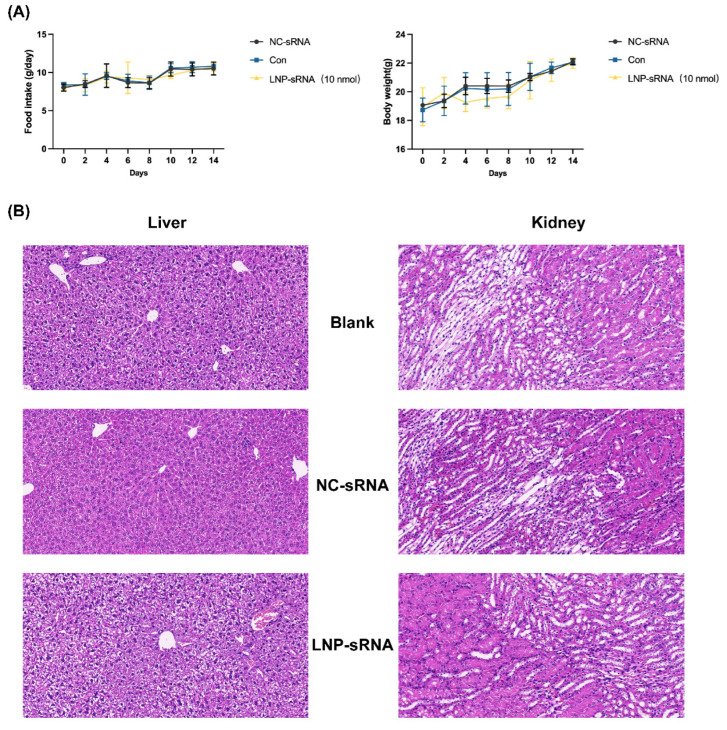
Safety evaluation of LNP-sRNA in vivo. (**A**). Physiological monitoring of mice during 14-day treatment with NC-sRNA, Control (Con), or LNP-sRNA (10 nmol). Food intake monitoring showing no significant differences among treatment groups. Body weight monitoring showing normal weight gain patterns across all groups, indicating no adverse systemic effects of treatments. (**B**). Histopathological evaluation of major organs after 14-day treatment. Representative H&E-stained sections of liver and kidney. No significant histopathological abnormalities, inflammatory infiltrates, necrosis, or structural changes were observed in the liver or kidney tissues of LNP-sRNA-treated mice compared to control groups, demonstrating the safety and biocompatibility of the optimized LNP-sRNA formulation. Data are presented as mean ± SEM.

Comprehensive safety evaluation (Figure 6) demonstrated that LNP-sRNA treatment did not adversely affect physiological parameters such as food intake or body weight over a 14-day treatment period. There were no observed abnormal adverse reactions and deaths occurred. Histopathological examination of major organs (liver and kidney) revealed no significant abnormalities, inflammatory infiltrates, necrosis, or structural changes in LNP-sRNA-treated mice compared to control groups. This favorable safety profile can be attributed to the biodegradable nature of the lipid components, the optimal particle size reducing nonspecific uptake, and the selective tissue distribution profile of our formulation [22].

## 4. Discussion

The development of effective therapeutic strategies for osteoporosis remains a critical challenge due to the limitations of current treatments, including adverse effects and transient efficacy. This study presents a novel LNP-based sRNA delivery system optimized for bone-targeted therapy, demonstrating significant improvements in osteogenic activity, biodistribution, and therapeutic efficacy in a preclinical osteoporosis model. Our findings highlight the potential of LNP-mediated sRNA delivery to address key barriers in osteoporosis treatment, offering a dual-action mechanism that simultaneously enhances bone formation and suppresses excessive resorption.

Current anti-resorptive agents and anabolic drugs often exhibit narrow therapeutic windows, systemic toxicity, or transient efficacy. Our optimized LNP-sRNA formulation demonstrated delivery of sRNA to bone tissue, with detectable levels maintained for up to 24 h. The delivery to bone tissue was enhanced compared to free sRNA administration, suggesting that LNP encapsulation improves overall bioavailability of the sRNA. The therapeutic efficacy observed in our osteoporosis model indicates functional delivery of sufficient sRNA to exert biological effects.

Previous approaches for bone-targeted drug delivery have primarily relied on bisphosphonate conjugation or hydroxyapatite-binding peptides to achieve skeletal targeting [26]. Our study demonstrates that carefully optimized LNP formulations can achieve appreciable bone accumulation without the need for additional targeting moieties, simplifying manufacturing and potentially reducing immunogenicity concerns.

The delivery of our LNP formulation to bone tissue presents an interesting scientific question when considered in light of recent literature. Martin et al. [28] demonstrated that bone marrow vessels are permeable primarily to nanoparticles smaller than ~30 nm, which suggests limitations for bone delivery. In our sequential optimization process, we initially identified formulations with N/P ratio of 8:1 showing good delivery properties despite larger sizes (208 nm), but through subsequent optimization of DMG-PEG2000 ratio and flow rate parameters, our final optimized formulation achieved a significantly reduced size (85.4 nm). However, our particles remain larger than the optimal size (<30 nm) suggested by Martin et al. for direct extravasation into bone tissue. The observed efficacy might be explained by accumulation of LNPs in bone vasculature with subsequent local release of the smaller sRNA molecules that can diffuse to target cells. Future formulation development could further explore size reduction strategies while maintaining the high encapsulation efficiency and stability achieved in our current formulation.

Our findings regarding the optimal N/P ratio (8:1) and PEG density (2.5% DMG-PEG2000) provide valuable insights for delivery to bone issues. These parameters significantly influence the in vivo behavior of LNPs by affecting surface charge, stability, and protein corona formation—all critical factors determining tissue distribution and cellular uptake [36]. Our study demonstrates that carefully optimized LNP formulations can achieve appreciable bone accumulation through intrinsic properties, simplifying manufacturing and potentially reducing immunogenicity concerns associated with additional targeting moieties.

Our data suggest that sRNA-OP-5, which targets secreted frizzled related protein 1 (SFRP1), exerts its therapeutic effects primarily through modulation of the Wnt signaling pathway. SFRP1 is an antagonist of Wnt signaling that competitively binds to Wnt ligands, preventing their interaction with Frizzled receptors [37]. By inhibiting SFRP1 expression, our sRNA enhances canonical Wnt signaling, which directly promotes osteoblast differentiation and function while indirectly inhibiting osteoclastogenesis through increased osteoprotegerin production [38]. This dual-action mechanism explains our observation of simultaneously reduced bone resorption marker and elevated bone formation marker levels (Figure 4C). Furthermore, enhanced Wnt signaling is known to suppress adipogenic differentiation of mesenchymal stem cells while promoting their commitment to the osteoblast lineage [39], consistent with our in vitro findings of increased osteogenic and reduced adipogenic differentiation (Figure 1). Future studies employing RNA-seq and pathway analysis will further elucidate the downstream molecular targets affected by SFRP1 inhibition in our system.

The observed osteogenic effects of LNP-sRNA may stem from its ability to modulate mesenchymal stem cell differentiation, as evidenced by reduced marrow adiposity and enhanced mineralization in treated mice. This suggests that sRNA-OP-5 targets pathways regulating lineage commitment, such as Wnt/β-catenin or BMP signaling, which are critical for osteoblastogenesis. The dose-dependent efficacy, even at low doses (1 nmol/kg), underscores the high potency of this platform, potentially reducing treatment frequency and improving patient compliance. Moreover, the absence of systemic toxicity—evidenced by stable body weight, normal organ histology, and negligible cytotoxicity in vitro—supports the translational potential of LNP-sRNA.

Our LNP-sRNA approach offers several advantages compared to other emerging osteoporosis therapeutics. Unlike Romosozumab (anti-sclerostin antibody), which has shown efficacy but raised cardiovascular safety concerns [40], our formulation demonstrated a favorable safety profile with no observed adverse effects on vital organs. Additionally, the flexibility of sRNA in targeting virtually any disease-relevant gene expands the potential therapeutic applications beyond what is possible with antibody-based approaches.

Compared to other RNA-based therapeutic strategies for osteoporosis, such as miRNA inhibitors or mRNA delivery, sRNA offers advantages in terms of potency, stability, and specificity [41]. The relatively short half-life of sRNA (days to weeks) compared to DNA-based gene therapies may actually be advantageous for osteoporosis treatment, allowing better dose titration and reducing long-term safety concerns associated with permanent genetic modifications.

The promising efficacy and safety profile of our LNP-sRNA formulation support its potential for clinical translation in osteoporosis treatment. The dose-dependent response observed in our study allows flexibility in clinical dose selection, with even the lowest tested dose showing significant therapeutic effects. And the IV administration route is clinically established and would be acceptable for a chronic condition like osteoporosis, particularly if dosing frequency can be limited to monthly or quarterly administration based on the observed pharmacokinetic profile.

While this study demonstrates promising results, several limitations warrant consideration. First, the osteoporosis model employed here primarily mimics postmenopausal bone loss; additional models such as aging- or glucocorticoid-induced osteoporosis should be explored to validate broader applicability. Second, long-term safety and immunogenicity assessments beyond 5 weeks are needed to ensure clinical viability. Finally, the precise molecular targets of sRNA-OP-5 and its effects on osteoclast activity require further elucidation. Future studies should investigate combinatorial strategies, such as co-delivering anti-resorptive and anabolic sRNAs, to maximize therapeutic outcomes. In addition, while our optimized LNP formulation achieved significant bone accumulation, the overall delivery efficiency still leaves room for improvement.

## 5. Conclusions

This study demonstrates the successful development of an optimized LNP formulation for efficient delivery of therapeutic sRNA to bone tissue, resulting in significant improvements in bone quality and strength in an osteoporosis model. The dual action of enhancing bone formation while reducing bone resorption, coupled with the favorable safety profile, suggests potential advantages over existing osteoporosis therapies. Future studies should focus on evaluating long-term efficacy and safety in larger animal models to further enhance therapeutic outcomes.

## Data Availability

The datasets used and/or analyzed during the current study are available from the corresponding author upon reasonable request.

## Supplementary Materials

The following supporting information can be downloaded at: https://www.mdpi.com/article/10.3390/pharmaceutics17060789/s1, Figure S1: sRNA-OP-5 inhibits adipogenic differentiation; Table S1: Sequences of primers; Table S2: LNP physicochemical properties with different nitrogen/phosphorus ratio; Table S3: LNP properties with different N/P ratio and DMG-PEG2000 percentage; Table S4: LNP physicochemical properties with different flow rate ratio; Table S5: Stability of LNP-sRNA formulations.
