# Supplementary material for: Intravenous Administration of sRNA Nanoparticles for Treatment of Osteoporosis in Mice
Source: Pharmaceutics. 2025 Jun 17;17(6):789. doi: 10.3390/pharmaceutics17060789 (PMC12196915; doi:10.3390/pharmaceutics17060789)
Supplement: Supplementary file 1 [file pharmaceutics-17-00789-s001.zip › Supplementary Figure S1.pdf]

Supplementary Figure 1

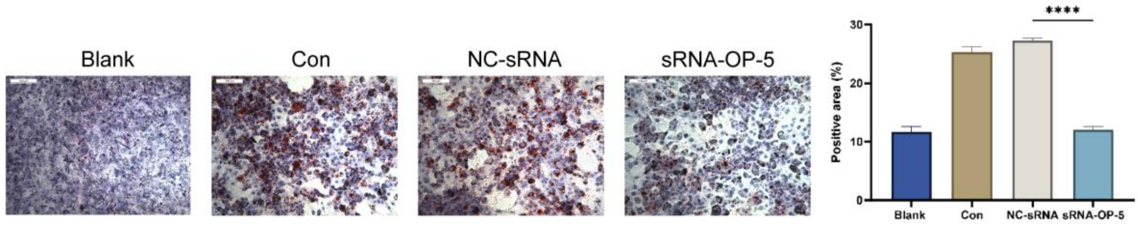

Supplementary Figure S1. sRNA-OP-5 inhibits adipogenic differentiation. Oil Red O staining and quantification of positive staining area demonstrate the inhibitory effect of sRNA-OP-5 on adipogenic differentiation compared to control conditions. Data are presented as mean ± SEM. **** $P < 0.000$.
