# Supplementary material for: Intravenous Administration of sRNA Nanoparticles for Treatment of Osteoporosis in Mice
Source: Pharmaceutics. 2025 Jun 17;17(6):789. doi: 10.3390/pharmaceutics17060789 (PMC12196915; doi:10.3390/pharmaceutics17060789)
Supplement: Supplementary file 1 [file pharmaceutics-17-00789-s001.zip › Supplementary Table.pdf]

Supplementary Table S1. Sequences of primers

**Name of primers**	**Primer sequence (5' to 3')**
Mouse Alpl Forward primer	CCAACTCTTTTGTGCCAGAGA
Mouse Alpl Reverse primer	GGCTACATTGGTGTTGAGCTTTT
Mouse Bglap Forward primer	TTCTGCTCACTCTGCTGACC
Mouse Bglap Reverse primer	GGGACTGAGGCTCCAAGGTA
Mouse Sp7 Forward primer	ATGGCGTCCTCTCTGCTTG
Mouse Sp7 Reverse primer	TGAAAGGTCAGCGTATGGCTT
Mouse Gapdh Forward primer	CACTCACGGCAAATTCAACGGCAC
Mouse Gapdh Reverse primer	GACTCCACGACATACTCAGCAC

Supplementary Table S2. LNP physicochemical properties with different nitrogen/phosphorus ratio

**Nitrogen/phosphorus ratio (N/P)**	**Size (d.nm)**	**PDI**	**Potential (mV)**	**EE%**
6	201.5	0.22	0.67	74.5%
8	208.6	0.22	1.10	75.0%
10	167.6	0.18	1.43	67.8%

Supplementary Table S3. LNP properties with different N/P ratio and DMG-PEG2000 percentage

**N/P**	**DMG-PEG2000**	**Size (d.nm)**	**EE%**
6	1.5%	98.3	85.5%
6	2.5%	85.4	81.1%
8	1.5%	110.1	91.5%
8	2.5%	93.2	93.4%

Supplementary Table S4. LNP physicochemical properties with different flow rate ratio

**Flow rate ratio (FRR) (mL/min)**	**Size (d.nm)**	**PDI**	**EE%**
8	106.5	0.15	94.4%
12	88.9	0.10	89.1%

Supplementary Table S5. Stability of LNP-sRNA formulations

Component	Time	Size (d.nm)	PDI
1	Day1	98.3	0.138
	Day7	100.2	0.103
2	Day1	85.4	0.201
	Day7	89.5	0.160

(Component 2 was our optimized formulation, and Component 1 was another formulation with 1.5% DMG-PEG2000.)
